# Supplementary material for: Age-associated changes of cytochrome P450 and related phase-2 gene/proteins in livers of rats
Source: PeerJ. 2019 Aug 2;7:e7429. doi: 10.7717/peerj.7429 (PMC6681801; doi:10.7717/peerj.7429)
Supplement: Table S1 [file peerj-07-7429-s002.docx]

Table 1. Primer sequence list.

| Gene | Accession^#^ | Sequence (5'‑3') | | Product length  (bp) |
| --- | --- | --- | --- | --- |
|  |  | Forward primer | Reverse primer |  |
| β-actin | NM_031144.3 | GGAGATTACTGCCCTGGCTCCTA | GACTCATCGTACTCCTGCTTGCTG | 150 |
| Cyp1a1 | NM_012540.2 | CATTGTGCCTGCCTCCTACTT | GTTCCTGTGGGTCTCTGCTGT | 81 |
| Cyp1a2 | NM_012541.3 | CACAGCACAACGAGGGACA | TCTGGGCGGAACACAAAG | 128 |
| Cyp2b1 | NM_001134844.1 | GGGAAAGAGGAGTGTGGAAGAA | GAGCAGATGATGTTGGCTGTG | 132 |
| Cyp2b2 | NM_001198676.1 | GGGAAAGAGGAGTGTGGAAGAA | GGAGAATGAACTTAGGAGGGAAAAG | 229 |
| Cyp2c6 | NM_001013904.1 | CTGTTGCTCCTGCTGAAGTGTCC | CCGCATGTGGCAGGTTGGTAG | 181 |
| Cyp2c11 | NM_019184.2 | AGCTTGGTGGCTACTGTAACTGAC | CAGCAGCAGCAGGAGTCCATAC | 87 |
| Cyp2d2 | NM_012730.1 | GCCTGGAAGCCTGTAGTTGTGATC | TCGTCTCTGCTCTCGCCACTC | 180 |
| Cyp2e1 | NM_031543.1 | GGAAGGATGTGCGGAGGTT | CAGAAATGTGGGGTCAAAAGG | 149 |
| Cyp3a1 | NM_013105.2 | ATGATTCCATCTTATGCTCTTCACC | CTGCCCTTGTTCTCCTTGCT | 95 |
| Cyp3a2 | NM_153312.2 | GATCCTTTTGTGGAGAAAACCAAG | TTGGGGTGAGGAATGGAAAG | 94 |
| Cyp4a1 | NM_175837.1 | CCACTCATTCCTGCCCTTCT | GCCACAATCACCTTCATCTCAC | 84 |
| Cyp7a1 | NM_012942.2 | AACCTGTGTGTGAGGGACCAG | TTGCTTGAGATGCCCAGAGA | 130 |
| Cyp8b1 | NM_031241.1 | GCGATGAAGGCTGTGCGAGAG | GTCTCTTCCATCACGCTGTCCAG | 131 |
| Cyp7b1 | NM_019138.1 | GAAGCTATGGAAGTCCTGCGTGAC | GGAGCACAACCTCAGAACCTCAAG | 156 |
| Cyp27a1 | NM_178847.3 | TCGCACCAATGTGAATCTGGCTAG | CTTCCACTGCTCCATGCTGTCTC | 97 |
| AhR | NM_013149.2 | GCTGGATAATTCATCTGGTTTCCT | GTCTTTCCCTTTCTTGTTCTGTCC | 85 |
| CAR | NM_022941.4 | GCATTGGATTGGAAAGGGTAAA | GATGACCGCACGAAGAGACA | 111 |
| PXR | NM_052980.2 | ATCCCCACCTCAGAAGACAAAG | AGAACCCCAGACCCTACACAAA | 127 |
| PPARα | NM_013196.1 | TTGCTGAAGTACGGTGTGTATGAA | TTAGGAACTCTCGGGTGATGAAG | 112 |
| FXR | NM_021745.1 | CAGCCACAGATCTCCTCCTC | TCTTTGTCACAGGCATCTCG | 160 |
| Ugt1a1 | NM_012683.2 | ACACCGGAACTAGACCATCG | TTGGAACCCCATTGCATATT | 153 |
| Ugt1a2 | NM_201423.2 | GGCTTCGAACCACAACATTT | GCTGCACAAGAATTTGCGTA | 92 |
| Sult1a1 | NM_031834.1 | TTTGCAGAGACAATTGGGCC | GCCACACTTCTCTAGCTTGC | 144 |
| Sult1a2 | NM_031732.2 | AACTTCCAAGCAAAGCCTGA | AACATCCCCATCATTTTGGA | 105 |
|  |  |  |  |  |
