# Supplementary material for: Age-associated changes of cytochrome P450 and related phase-2 gene/proteins in livers of rats
Source: PeerJ. 2019 Aug 2;7:e7429. doi: 10.7717/peerj.7429 (PMC6681801; doi:10.7717/peerj.7429)
Supplement: Table S2 [file peerj-07-7429-s003.docx]

Supplemental table 2 Protein expression of CYP1A2, CYP2B1, CYP2E1, CYP3A1, CYP4A1 and CYP7A1.

| Gene | 14 d | 21 d | 28 d | 60 d | 180 d | 540 d | 800 d |
| --- | --- | --- | --- | --- | --- | --- | --- |
| CYP1A2 | 1.0 ± 0.0 | 1.42 ± 0.16 | 1.83 ± 0.10 | 1.765 ± 0.10 | 1.83 ± 0.21 | 1.75 ± 0.22 | 1.816 ± 0.14 |
| CYP2B1 | 1.0 ± 0.0 | 1.15 ± 0.17 | 0.85 ± 0.14 | 0.81 ± 0.13 | 0.68 ± 0.18 | 0.52 ± 0.13 | 0.51 ± 0.12 |
| CYP2E1 | 1.0 ± 0.0 | 1.03 ± 0.12 | 1.15 ± 0.13 | 1.55 ± 0.21 | 1.48 ± 0.16 | 1.14 ± 0.12 | 1.08 ± 0.15 |
| CYP3A1 | 1.0 ± 0.0 | 1.48 ± 0.16 | 1.75 ± 0.17 | 1.57 ± 0.22 | 1.46 ± 0.19 | 1.25 ± 0.16 | 1.18 ± 0.11 |
| CYP4A1 | 1.0 ± 0.0 | 1.06 ± 0.13 | 1.42 ± 0.15 | 1.32 ± 0.10 | 1.31 ± 0.13 | 1.28 ± 0.14 | 1.25 ± 0.15 |
| CYP7A1 | 1.0 ± 0.0 | 1.23 ± 0.17 | 1.52 ± 0.17 | 1.65 ± 0.22 | 1.66 ± 0.25 | 1.50 ± 0.12 | 1.24 ± 0.11 |
